# An Overview of Circulating Cell-Free Nucleic Acids in Diagnosis and Prognosis of Triple-Negative Breast Cancer

**DOI:** 10.3390/ijms24021799

**Published:** 2023-01-16

**Authors:** Domenico Tierno, Gabriele Grassi, Fabrizio Zanconati, Marina Bortul, Bruna Scaggiante

**Affiliations:** 1 Department of Life Sciences, University of Trieste, 34127 Trieste, Italy; 2 Department of Medical and Surgical Sciences, Hospital of Cattinara, University of Trieste, 34149 Trieste, Italy

**Keywords:** TNBC, circulating cell-free nucleic acids, liquid biopsy, circulating cell-free tumor DNA, circulating cell-free tumor miRNA, circulating cell-free tumor lncRNA

## Abstract

Triple-negative breast cancer (TNBC) is an aggressive subtype of breast cancer due to its molecular heterogeneity and poor clinical outcomes. Analysis of circulating cell-free tumor nucleic acids (ctNAs) can improve our understanding of TNBC and provide efficient and non-invasive clinical biomarkers that may be representative of tumor heterogeneity. In this review, we summarize the potential of ctNAs to aid TNBC diagnosis and prognosis. For example, tumor fraction of circulating cell-free DNA (TFx) may be useful for molecular prognosis of TNBC: high TFx levels after neoadjuvant chemotherapy have been associated with shorter progression-free survival and relapse-free survival. Mutations and copy number variations of TP53 and PIK3CA/AKT genes in plasma may be important markers of TNBC onset, progression, metastasis, and for clinical follow-up. In contrast, the expression profile of circulating cell-free tumor non-coding RNAs (ctncRNAs) can be predictive of molecular subtypes of breast cancer and thus aid in the identification of TBNC. Finally, dysregulation of some circulating cell-free tumor miRNAs (miR17, miR19a, miR19b, miR25, miR93, miR105, miR199a) may have a predictive value for chemotherapy resistance. In conclusion, a growing number of efforts are highlighting the potential of ctNAs for future clinical applications in the diagnosis, prognosis, and follow-up of TNBC.

## 1. Clinical and Molecular Characterization of Triple-Negative Breast Cancer

Female breast cancer is the most commonly diagnosed neoplasm worldwide. The International Agency for Research on Cancer (IARC) report from 185 countries worldwide estimates that 2.3 million new cases (11.7%) of female breast cancer occurred in 2020, compared with 2.2 million new cases of lung cancer (the second most commonly diagnosed tumor) [1]. Incidence rates are higher in countries with a high Human Development Index (HDI) than in countries with a low HDI. This is due to the fact that in HDI countries, mammography screening is more efficient and lifestyle risk factors (such as alcohol abuse and obesity) are more common. Furthermore, breast cancer is the fifth leading cause of tumor-related death in women worldwide, with approximately 685,000 deaths reported in 2020 [1]. According to the international consensus guidelines [2,3], breast cancer can be classified into four molecular subtypes defined by histological analysis and immunohistochemical analysis of progesterone receptor (PR), estrogen receptor (ER), and human epidermal growth factor receptor 2 (HER2). Luminal A is a low-grade breast cancer subtype with ER/PR positivity and the absence of HER2. In contrast, Luminal B is positive for ER, but expression levels of HER2/PR are variable. HER2+ subtype is characterized by the expression of HER2 and the absence of PR/ER, whereas triple-negative breast cancer lacks all three receptors [2,3]. The different molecular behavior of breast cancer subtypes is related to the clinical response to treatments [4]. For this reason, the development of different clinical approaches tailored to each molecular subtype is of great importance.

Triple-negative breast cancer (TNBC) is a low-incidence subtype of breast cancer characterized by a high risk of metastasis and recurrence. According to the American Cancer Society, TNBC accounted for 10% of all breast cancers diagnosed in the United States between 2015 and 2019, with an incidence twice as high in black women as in white women [5]. The 5-year survival rate is 78.6%, decreasing to 54.2% and 26.1% when diagnosed at stage III and IV, respectively [6]. The lack of HER2, ER, and PR positivities hinders the efficacy of current hormonal and HER2-targeted therapies. In addition, the wide heterogeneity, aggressive behavior, and late diagnosis further limit the therapeutic options of TNBC [7]. These features, together with the lack of appropriate predictive and prognostic biomarkers, lead to a poor overall outcome [8]. Therefore, TNBC represents an important challenge for breast cancer research, and the discovery of efficient biomarkers is of great importance for clinical treatment.

Molecular characterization of TNBC may be useful to elucidate its heterogeneity and thus improve therapeutic approaches. Over the years, several molecular classifications of TNBC have been proposed, the most recent of which is based on the inclusion of mRNA and lncRNA transcriptome profiles (Fundan University Shangai Cancer Centre classification, FUSCC) [9,10]. Accordingly, four different subtypes with specific molecular and biological characteristics can be distinguished: immunomodulatory (IM), luminal androgen receptor (LAR), mesenchymal-like (MES), and basal-like and immunosuppressed (BLIS) [11,12]. Molecular subtyping may have relevant clinical implications: the subtype LAR, characterized by oncogenic activation of the ER pathway, responds to both anti-estrogen and anti-androgen therapies despite its ER negativity [9]. Beyond the FUCCS classification, the identification of specific molecular features paves the way to determine the most appropriate treatment. For example, TNBCs harboring BRCA1/2 mutations respond very well to PARP inhibitors (PARPi) due to their defective DNA repair machinery [13]. Indeed, BRCA1/2 genes are involved in the repair of double-strand damage, whereas PARP acts on single-strand damage. Therefore, inhibition of PARP in cells deficient in BRCA1/2 repair leads to the accumulation of single-strand damages and then to the formation of double-strand lesions [14]. Regarding TNBC with BRCA deficiency, the OlympiAD trial showed how PARPi Olaparib treatment resulted in significantly higher progression-free survival (PFS) compared to standard chemotherapy (capecitabine, eribulin mesylate or vinorelbine) [15,16]. In addition, several TNBCs have high levels of PD-L1 (cell death protein ligand 1) resulting in remarkable genomic instability and increased immune infiltration [9,17]. PD-L1 is frequently overexpressed on tumor cells and binds to its receptor PD-1 on the surface of T-cells to inhibit them [18]. These TNBCs are sensitive to PD-L1 inhibitors such as atezolizumab and pembrolizumab, whose clinical trials have shown promising results and FDA approval of atezolizumab for metastatic TNBC in 2019 [19,20]. Another molecular footprint of TNBC is the dysregulation of the PI3K/AKT pathway. Although mutations in single genes are relatively rare, combined activating mutations in PIK3CA and AKT1 occur in 25–30% of advanced TNBC [21]. This highlights the potential use of AKT inhibitors in combination with first-line chemotherapy to improve the clinical outcome of TNBC patients [22].

## 2. Liquid Biopsy and Circulating Cell-Free Nucleic Acids in TNBC

**Figure 1:**
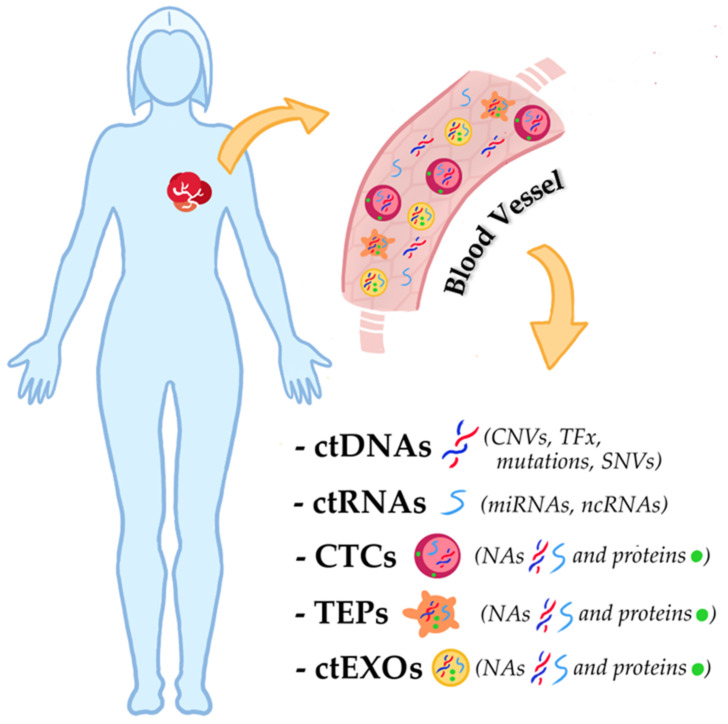
Scheme of principal targets from liquid biopsy in TNBC research. As shown in the figure, the biomolecular analysis from body fluids, such as blood, is not limited to circulating cell-free nucleic acids but also considers the tumor biomolecules carried by CTCs, TEPs, and ctEXOs. Abbreviations: CNVs (copy number variations); CTCs (circulating tumor cells); ctDNAs (circulating cell-free tumor DNAs); ctEXOs (circulating tumor exosomes); ctncRNAs (circulating cell-free tumor non-coding RNAs); NAs (nucleic acids); ncRNAs (non-coding RNAs); SNVs (single nucleotide variations); TEPs (tumor-educated platelets); TFx (tumor fraction of circulating cell-free DNA).

Considering the potential of molecular studies for the clinical treatment of TNBC, circulating cell-free tumor nucleic acids (ctNAs) represent a promising field of research. The most important advance in the use of ctNAs in routine clinical practice is related to the possibility of obtaining them from liquid biopsies [23]. The latter refers to various biological fluids from patients, such as blood or urine, from which recovered circulating tumor cells (CTCs), circulating cell-free nucleic acids (ccfNAs), exosomes, and “tumor-educated platelets” (TEPs). The analysis of these biological components allows the detection, and real-time monitoring of tumors through non-invasive procedures [24]. CTCs, ctNAs, TEPs and exosomes are indeed representative of the heterogeneity of the primary tumor or metastases and can therefore be used as informative biomarkers. CTCs are cells that detach from the tumor and migrate through the bloodstream. Despite their low abundance (<10 cells/mL), isolation and characterization of CTCs can provide histological, molecular, and biological information about the tumor [25,26]. TEPs are platelets that have directly taken up circulating mRNA released by tumor cells and can also undergo queue-specific splicing events in response to signals released by cancer cells and the tumor microenvironment, such as stromal and immune cells. TEPs are also capable of sequestering solubilized tumor-associated proteins and for this reason, can serve as markers of tumor progression [27]. Exosomes are a class of extracellular vesicles that contain proteins and nucleic acids of the cells from which they are released. They are detectable in the blood of patients with different types of cancers and carry various biomolecules from tumor cells. For this reason, the characterization of the molecular content of exosomes can provide useful clues about the original tumor cells [28,29]. Ultimately, ctNA is the fraction of the ccfNA derived from the tumor. It can be DNA (ctDNA), mRNA (ctmRNA), miRNA (ctmiRNA), or other non-coding RNA (ctncRNA), all of which have potential applications in clinical research. For example, ctDNA changes and concentrations are associated with various pathological features, such as mutational burden, the presence of metastasis, and the response to biological anti-tumor drugs [30]. In addition, the integrity of circulating cell-free DNA could be a useful diagnostic and prognostic marker (cfDI) [31]. Finally, ctmiRNA may be an efficient predictive and prognostic biomarker for various tumors, as miRNAs are involved in many tumor processes, such as tumor growth and chemoresistance, and are specifically related to tumor subtypes [32]. The principal targets from liquid biopsy in TNBC research are shown in Figure 1.

Although ctNAs are detectable in many biological fluids (urine, cerebrospinal fluid, saliva, and pleural fluid depending on tumor type), they are usually highly fragmented and present at low concentrations in liquid biopsies [33,34]. To enable the efficient clinical application of ctNAs, it is to achieve an acceptable level of ctNAs purification and good sensitivity and specificity of techniques for analysis. From this point of view, the development and optimization of various workflows to maintain the ccfNA integrity and of technological platforms, such as digital droplet PCR or new-generation sequencing techniques, have greatly improved the possibilities of using ctNAs in routine clinical practice [35]. Accordingly, this review aims to summarize the efforts on circulating tumor nucleic acids (ctNAs) with clinical relevance in TNBC (2017–2022 peer-reviewed articles in PubMed).

### 2.1. Circulating Cell-Free Tumor DNA in TNBC

In the last 5 years, the efforts on ctDNAs in TNBC focused on the evaluation of their predictive and prognostic value. A prominent role in TNBC progression and treatment was given at two important ctDNA parameters: the mutant allele frequencies (MAF) or variant allele frequencies (VAF) and the ctDNA fraction (or tumor fraction, TFx). MAF/VAF refers to the relative frequencies of one or more mutant alleles in a population [36]. TFx indicates the ratio between ctDNA fraction and the total circulating cell-free DNA (ccfDNA) [37]. Entity and changes of both parameters are an expression of tumor progression events and for this reason, can be used as cancer biomarkers [36,37]. An example of this is given by the efforts of Cailleux et al., which have analyzed the plasma samples of 13 TNBC, 11 HER2+, and 20 ER+ breast cancer patients at different time points: pre-NAC (baseline) and post-NAC treatments, before surgery and after surgery [38]. They have performed ctDNA detection through a personalized and tumor-informed assay (bespoke multiplex polymerase chain reaction NGS ctDNA assay, Signatera). This assay allows the selection of a personalized tumor-informed DNA variant panel for each patient and analyzes it on the plasma sample. The analysis has shown a higher VAF at baseline in TNBC patients compared to HER+ and ER+ ones. Highly aggressive and proliferative tumors are indeed associated with a high ctDNA detection rate [39]. Moreover, they have noted a shorter event-free survival for patients that displayed a high VAF at post-NAC and beyond time points [38]. Park et al. emphasize the clinical importance of cell-free circulating DNA concentration also with a study aimed to assess this parameter as an early predictive biomarker of relapse in TNBC patients [40]. They performed a quantification fluorescence assay (The SYBR Gold Nucleic Acid Gel Stain) on plasma samples of 72 TNBC patients before chemotherapy (baseline) and after four cycles of Adriamycin plus cyclophosphamide. They have noted a significant decrease in average cfDNA concentration after treatment for most patients. Despite this, the analysis has not shown a correlation between ccfDNA variation and radiologic or pathologic response to NAC. According to the authors’ opinion, the lack of correlation can be due to the relatively small sample size [40]. However, this analysis has identified a ccfDNA threshold value (264 ng/mL) at baseline over which the relapse risk is higher. The authors suggest the ccfDNA concentration at baseline as an independent prognostic value of relapse risk in TNBC since it represents the systemic tumor burden, including metastasis or primary sites [40]. The report of Ortolan et al. on 31 TNBC patients pre- and post-NAC treatments represents proof of the predictive significance of ctDNA recognition after chemotherapy [41]. They performed an initial pre-NAC primary tumor-targeted gene sequencing (IonAmpliSeq Cancer Hotspot Panel v2 CHPv2) on each patient and a subsequent patient-specific point mutation ddPCR detection on plasma. This approach is useful to avoid false positives, but it is important to underline that eventual clonal evolutions cannot be studied. However, the analysis has shown that the presence of ctDNA post-NAC led to a shorter 2-year event-free survival. Moreover, the ctDNA predictive value remains strong also after adjusting the comparison for age, residual disease, systemic inflammatory indices, and Ki-6 [41]. Wongchenko et al. provided interesting hints on VAF variation during TNBC clinical management. They analyzed the plasma samples of 89 TNBC patients before (baseline) and during treatment with AKT inhibitor Ipatasertib plus paclitaxel [42]. They have used a narrow ctDNA panel targeting short variants identified in baseline samples by FoundationACT hybrid capture NGS assays (on blood samples) and FoundationOne hybrid capture NGS assays (on tissue biopsy) to quantify the variant fraction of mutations (VAF) in on-treatment samples. As expected, they have found a shorter progression-free survival in patients with high VAF during treatment [42]. Moreover, the analysis has shown a higher improvement in progression-free survival after treatment with ipatasertib plus paclitaxel for patients with PIK3CA/AKT mutations at baseline than those without. This result highlights one more time the importance of the AKT pathway in TNBC progression and treatment. However, the NGS assay used in this effort assesses only a limited number of genes and does not consider the copy number variations. Another important clinical implication of ctDNA concentration is suggested by Jacob et al. They have performed a Guardant360 next-generation sequencing (NGS) quantification assay on two different groups of metastatic breast cancer [43]. The first one is composed of 44 HR+ (hormone-positive), 20 HER2+, and 22 TNBC patients with one or more ctDNA collections. The first ctDNA collection was identified and defined as a baseline. Other two ctDNA collections during patient follow-up are considered for this group. The latter collections were related to evidence of clinical or radiological progression leading to change in treatment. They are indicated and defined as PN1 (post NGS progression 1) and PN2 (post NGS progression 2). An additional cohort of 62 patients (53 HR+, 2 HER2+, 6 TNBC) with several ctDNA testing and without radiographic disease progression evidence was used as the control group. The Guardant360 NGS assay is an FDA-approved test for a complete genomic profile that covers all genes recommended by the National Comprehensive Cancer Network [44]. This test considers for each gene both mutations (single nucleotide variations, insertions, deletions) and amplifications. The analysis has proved how at PN1 and PN2 the MAF increase in patients with tumor progression compared to the control ones without progression. These data have indicated the MAF increase as a potential biomarker of increasing genomic burden and tumor progression. Interestingly, the analysis of ctDNA at the different time points showed several likely resistance alterations, such as TP53, PI3KCA, AR, ESR1, ERBB2, and FGFR1 [43]. These genes are indeed involved in TNBC progression (TP53, PIK3CA), hormone-positive breast cancer (HR+), and endocrine therapy resistance (ESR1, ERBB2, FGFR1, AR) [45,46,47]. Lin et al. have achieved similar results on plasma samples from 95 breast cancer patients (among which 25 TNBC). In this report, the mutation and copy number state of a gene panel (TP53, PI3KCA, HER2, GATA3, CDH1, PTEN, AKT1, ESR1, S100A7-9, ZNF703, B2M, CCND1, c-MYC) were analyzed pre- and post-neoadjuvant chemotherapy (NAC) [48]. Li et al. used a personalized QIAseq Targeted DNA Panel to amplify the coding region of chosen genes. The resulting library has been sequenced by Illumina MiSeq Reagent Kit v2, 2 × 150 bp reads to analyze the pathogenic mutations. The copy number variation has been analyzed by OncoCNV. They found that the post-NAC detection of alterations in selected genes is associated with worse relapse-free survival. Among these patients, HER2+ cases have displayed a longer relapse-free survival than regards the TNBC ones, probably due to the efficiencies of maintenance anti-HER2 antibody therapy [48]. The importance of specific ctDNA alterations in TNBC progression is further highlighted by Cavallone et al. They analyzed tumor and serial blood samples from 26 TNBC patients collected before, during, and after NAC treatment by developing individual digital droplet PCR assays for 121 variants (average of five variants for a patient) previously identified from tumor genomic profiling through whole exome sequencing (WES) [49]. They selected the genes having variants according to three criteria: (a) genes with the highest MAF in tumors, (b) TP53 variants, and (c) genes with change in MAF before and after NAC in tumors. Moreover, they have set up a threshold of detection for each allele frequency of all variants investigated. To do this, they performed the personalized ddPCR assay on plasma samples of 30 healthy volunteers. The use of a 4–5 tumor-informed variants panel has enabled ctDNA detection in 96% of patients at baseline. The analysis has shown that the detection of ctDNAs during NAC is strongly predictive of tumor residual after surgery. Moreover, the detection of chosen variants after NAC has been related to poor overall survival and relapse-free survival [49]. To conclude, all the reports described so far pointed the light on the potent value of ctDNA detection after NAC as a prognostic biomarker of relapse risk and clinical outcome in TNBC. The ctDNA reflects indeed the tumor burden and their increase in liquid fluids may predict tumor progression events.

TNBC is characterized by a high gene copy number alterations rate [50] and so the ctDNA investigation of this parameter has a high clinical value. The amplification of 17q22 has been already described in breast cancer [51] and its pro-cisplatin consequence can be related to the overexpression of some genes in this region such as KIF2B, TRIM37, NR1D1, and DLX4. The latter is involved in the G2/M cell cycle checkpoint and inhibition of DNA repair mechanisms and their overexpression can improve the TNBC sensitivity to DNA-damaging platinum chemotherapy [51,52,53,54]. Collier et al. provided an excellent example of chromosome alteration analysis in deepening the implication of 17q22 amplification in plasma samples of 58 TNBC patients treated with cisplatin alone or in combination with paclitaxel [55]. They proved how the patients that displayed 17q22 amplification had better progression-free survival than the other ones after cisplatin treatment [55]. Instead, Stover et al. suggested a possible use of ctDNA amplification analysis as a metastatic biomarker. They performed a low-cover whole genomic profile on ccfDNA from 164 metastatic TNBC patients [56]. Subsequently, the sequencing data have been analyzed by IchorCNA, an R tool for the estimation of tumor fractions in ultra-low pass whole genome sequencing (WGS) and prediction of large-scale copy number variation (CNV). This approach allows the evaluation of ccfDNA tumor fraction without a priori tumor mutation status. In this way, the authors found that a ccfDNA tumor fraction over 10% is a marker of worse OS, reinforcing the results previously reported [56]. Among the analyzed cohort, Stover et al. identified 20 patients whose primary tumor underwent a target panel sequencing as part of clinical management. Subsequently, they compared the frequency of copy number variations for 25 genes commonly altered in breast cancer cfDNA between metastatic and paired primary tumors. Stover et al. have seen a higher amplification rate for AKT2, AKT3, and NOTCH2 genes in metastasis compared to paired primary tumors and primary TNBCs in publicly available data, The Cancer Genome Atlas, and METABRIC [56].

The validation of liquid biopsy ctDNA mutations in TNBC tissues has been provided by Wongchenko [42] and Jacob [43] efforts previously cited. In particular, Wongchenko et al. performed a molecular characterization in either liquid biopsy or primary/metastatic tumor tissues of 89 TNBC patients enrolled in the LOTUS clinical trial. They found a 100% concordance between ctDNA and genomic DNA tissue profiles in patients for activating PIK3CA or AKT1 mutations. Interestingly, the origin of tissue samples (metastatic or primary) does not seem to influence the concordance percentage [42]. This suggests PIK3CA and AKT mutations are possible early TNBC genetic events in primary tumors that are maintained during metastasis. Jacob et al. highlighted the high correlation between ctDNA and genomic DNA mutations also. They analyzed in a retrospective study, the molecular profile of plasma and tumor biopsy from 86 metastatic breast cancers, among which were 22 TNBC cases. They found that alterations in TP53, PIK3CA, ERBB2, EGFR, ESR1, CCNE1, MYC, NF1, MET, and KIT showed a high concordance (around 80% for each gene) between plasma and tissues. Since most of the tissue samples were taken from metastatic sites, it has been suggested that these alterations may arise early in tumor progression [43].

As described so far, the efforts on ctDNA involved in TNBC are focused on the ones achieved from blood samples. Herzong et al. suggested urine as an alternative liquid biopsy fluid from which to extract ctDNAs for TNBC clinical analysis. They compared the performances of ctDNA collection and characterization in both urine and plasma samples from 15 presurgical TNBC patients [57]. The analysis was performed by amplification of a coding region of 93 genes, known to be related to breast cancer, and their sequencing by Illumina NGS (NextSeqv2.5High Output 300 bp cassette). The comparison showed a higher ctDNA concentration in urine than plasma, but there was no significant correlation between matched samples. Bioinformatical analysis has revealed 431 somatic breast cancer-related variants shared in both fluids. Among these, the most common pathogenic and probable pathogenic mutations are NF1, CHEK2, KMT2C, and PTEN, which are tumor suppressor genes commonly implicated in TNBC onset and progression [43,44,45,46,47,48,57]. These results proved that both plasma and urine-derived ctDNA from TNBC patients could be efficiently analyzed using a targeted sequencing approach. Both body fluids appear to be valuable sources containing complementary information regarding the genetic tumor profile, that may be relevant to the clinical management of the disease. Despite the small size of the study cohort, this explorative report paves the need to further investigation of the clinical value of urinary ctDNA. Indeed, urine collection is a less invasive and more patient-friendly procedure than venous blood sampling [58]. 

The main findings from the literature cited in this chapter are summarized in Table 1.

### 2.2. Circulating Cell-Free Tumor Non-Coding RNA in TNBC

Non-coding RNAs (ncRNA) are a group of functional nucleic acids that are not translated into proteins. They represent the majority of the transcribed RNAs: protein-coding mRNAs account for only 3% of the total [59]. Depending on their length, shape, and localization, several different classes can be distinguished, each one has a specific biological function [60]. The most abundant and studied ncRNAs are microRNAs (miRNAs), long ncRNAs (lncRNA), circular RNAs (circRNAs), and PIWI-interacting RNAs (piRNAs). The main role of ncRNAs is to regulate gene transcription and protein synthesis through mRNA degradation (miRNA, lncRNA, circRNA), modulation of transcription factors (lncRNA, circRNA), and epigenetic regulation (piRNA) [61]. Due to their involvement in cellular expression networks, ncRNAs play a critical role in biological processes, and their aberrant dysregulation led to various pathological conditions such as neoplasms [61,62].

Like DNA, tumor non-coding RNAs (tncRNAs) can also be detected in biological fluids [63], and for this reason, several efforts have been made to use them in clinical cancer management. The circulating tumor miRNAs (ctmiRNA) are by far the class of tncRNAs with the most effort and finding in the literature. MiRNAs are small single-stranded ncRNAs (19–23 bases) involved in eukaryote gene regulation and are frequently associated with tumor stage, metastasis, and chemotherapy resistance [32]. Regarding TNBC, most studies have focused on the evaluation of ctmiRNA expression profiles as a biomarker for breast cancer subtype differentiation. For example, Niedzwiechi et al. analyzed the expression of ctmiRNA-200c, ctmiRNA21, and ctmiRNA10b in 46 breast cancer patients (37 ER+/PR+ and 9 TNBC) and found higher expression of miRNA 200c in ER+/PR+ patients than in TNBC patients. Interestingly, miRNA-200c acts as an oncogene regulating apoptosis, survival, and metastasis through PTEN inhibition and TP53 phosphorylation [64]. However, this result confirms the downregulation of miRNA-200c at the cellular level in TNBC patients, as reported by other works [65,66]. In a report by Qattan et al., the expression levels of 84 cancer-related miRNAs were examined in plasma samples from 34 healthy controls, 36 triple-negative, 16 luminal A, and 41 luminal B breast cancer patients before surgery and NAC. They reported higher expression of miR-19a, miR-19b, miR-25, miR-22, miR-93, miR-210, and lower expression of miR199a in TNBC compared to the other breast cancer subtypes [67]. These different expressions may have an impact on chemotherapy resistance. MiR19a has been shown to regulate anti-tumor immunity [65], while miR 19b actives NFkB and represses PTEN, which promotes cell proliferation and survival [68]. In addition, miR25 promotes cell proliferation via the AKT signaling pathway and inhibition of apoptosis [69], miR93 inhibits PTEN and is involved in TNBC progression and metastasis [70], while miR199a acts on mTOR and affects sensitivity to doxorubicin [71]. Indeed, Qattan et al. found that overexpression of miR-93, miR-210, miR19a, and miR19b was associated with significantly worse overall survival [67]. A contribution to this field was also made by Li et al. who focused on miR-105 and miR-93 plasma expression levels in both TNBC and non-TNBC patients. These miRNAs were selected from the METABRIC database after analyzing the miRNA expression profiles in TNBC and non-TNBC patients. They found higher expression of these ctmiRNAs in TNBC patients than regards the other breast cancer. Both miR-105 and miR-93 are involved in the activation of Wnt/B-catenin, leading to the promotion of stemness, chemoresistance, and metastasis [72]. Indeed, overexpression of miR-105 and miR93 is correlated with poor survival in TNBC patients. Finally, Triantafyllou et al. performed a comprehensive ctmiRNA characterization of HER2+, luminal A, luminal B, and TNBC. Specifically, they analyzed the miRNA expression using the miScript™ miRNA PCRArray Human Cancer Pathway Finder kit, and then applied a machine-learning approach to miRNA profiling for the different molecular subtypes of breast cancer [73]. Analysis showed that several miRNAs are differentially expressed in each breast cancer subtype (see Table 2). Regarding TNBC, they indicated overexpression of circulating miR-17, miR-133b, miR-210, miR-146b, and miR7 and under-expression of circulating miR-150, miR-372, and let-7f as the signature of TNBC. Among these, miR-17, miR-150, and miR-210 exhibit the best predictive properties. Of note, miR-17 is involved in cell proliferation [74], while the role of miR-150 and miR-210 is still debated in the literature [75].

An interesting contribution to ctmiRNA research was made by Ritter et al. who tested a personalized panel of TNBC-related miRNAs on serum and urine samples from 8 TNBC patients and 20 healthy controls [76]. The selected miRNAs (Let-7a; let-7e; miR-7, miR-9, miR-15a, miR-17, miR-18a, miR-19b, miR-21, miR-30b, miR-222, and miR-320c) came from an extensive literature search aimed at highlighting TNBC-related ncRNAs. As for blood samples, the analysis showed overexpression of Let7a, let7e, and 21b and under-expression of miR15a, miR17, miR18a, mir19b, and miR30b in TNBC patients compared with healthy controls. In contrast, in urine, TNBC patients showed under-expression of miR18b, miR19b, miR30b, miR222b, and miR320c compared with healthy controls. Unfortunately, the small sample size (8 patients) hinders the efficient clinical evaluation of these results [76]. Nevertheless, this report may provide further evidence for the ctmiRNA expression profile as a promising characterization biomarker.

Analysis of circulating cell-free ncRNAs in TNBC is not limited to ctmiRNA but also includes the study of other classes of ncRNAs, such as circulating long non-coding RNAs (clncRNA). The latter are non-coding transcripts usually longer than 200 nucleotides and are functionally involved in gene expression, subcellular transport, protein degradation, and organelle biogenesis [77]. Like miRNA, lncRNAs can also be associated with various cancer processes and can be obtained from biological fluids [78]. In the recent literature, there are some reports investigating circulating cell-free tumor lncRNAs as TNBC biomarkers. For example, Wang et al. performed a comprehensive analysis of TINCR expression in plasma samples from 72 TNBC, 105 non-TNBC, 60 benign breast disease, and 86 healthy controls. TINCR is an lncRNA that stimulates the expression of key differentiation genes [79]. Recently, aberrant expression of TINCR was shown to be associated with the progression of several types of cancer such as breast cancer and hepatocellular carcinoma [80]. Real-time PCR performed by Wang et al. revealed higher expression of this lncRNA for TNBC patients compared with non-TNBC, benign breast disease, and healthy individuals. The analysis also showed a negative correlation between TICR expression and overall survival/relapse-free survival in TNBC, but no correlation was observed in other breast cancer subtypes [81]. Instead, Bermejo et al. tested some promising cancer-related methylation probes on plasma samples from TNBC patients and healthy controls to identify epigenetic biomarkers. The analysis indicated hypermethylation of circulating LINC00299 as a marker for TNBC, especially for the age group of 26–52 years [82]. ChIP-seq data from ENCODE show that the hypermethylated region of LINC00299 is a non-coding functional element with binding sites for several transcription factors, such as RAD21, EP300, GATA2, and GATA3. These genes are involved in immune cell development, proliferation, and maintenance [83,84].

The main findings from the literature cited in this section are summarized in Table 2.

**Table 2:** Summary of TNBC clinical features, experimental method, and main results in liquid biopsy on ctRNA. Abbreviations: BBD (benign breast disease); ctlncRNA (circulating cell-free tumor lncRNA); ctmiRNA (circulating cell-free tumor miRNA); ER+ (estrogen receptor-positive breast cancer); HC (healthy controls); HER2+ (HER2- positive breast cancer); LumA (Luminal A breast cancer); LumB (Luminal B breast cancer); OS (overall survival); PR+ (progesterone receptor-positive breast cancer); TNBC (triple-negative breast cancer).

Number of Patients and Controls/Age	Clinical Features at Sample Collection (Number of Patients)	Source of ctRNA (Analyte)	Target	Method	**Main Findings**	**Ref.**
9 TNBC, 37 PR+/ER+ patients/TNBC: 56.48 yrs, PR+/ER+: 52.53 yrs.	All metastatic patients analyzed before surgery	Serum (ctmiRNA)	Expression of miR21, miR10b, miRNA-200c	qRT-PCR	Higher expression of miRNA 200c in ER+/PR+ patients than in TNBC ones.	[64]
36 TNBC, 16 LumA, 41 LumB, 34 HC/BC: 46 ± 10.55 yrs; HC: 29 ± 7.5 yrs	Metastatic (23), non-metastatic (70); all patients analyzed before surgery and therapy	Plasma (ctmiRNA)	Expression of 84 breast cancer-related miRNAs	MIHS-109Z miScript miRNA Array Human panel, qRT-PCR	miR19a, miR19b, miR93, miR25, miR22 and miR210 have a higher expression in TNBC than lumA/B cancers and HC. MiR199a is under-expressed in TNBC than LumA/B and HCs. The overexpression of miR-93, miR-210, miR19a, and miR19b is associated with significantly worse OS.	[67]
74 TNBC, 44 non-TNBC, 12 HC	All patients analyzed during NAC	Plasma (ctmiRNA)	Expression of miR93 and miR105	qRT-PCR	miR93 and miR105 have a higher expression in TNBC than in non-TNBC patients. Overexpression of miR93 and miR105 is correlated with poor survival in TNBC patients.	[72]
8 TNBC, 20 HC/TNBC: 55.4 yrs HC: 49 yrs	All metastatic patients analyzed before and during NAC	Serum; urine (ctmiRNA)	Expression of Let-7a; let-7e; miR-7, miR-9, miR-15a, miR-17, miR-18a, miR-19b, miR-21, miR-30b, miR-222 and miR-320c	qRT-PCR	Overexpression of let7a, let7e, miR21B and under-expression of miR15a, miR17, miR18a, miR19b, miR30b in TNBC serum compared to HC serum. Under-expression of miR18a, miR19b, miR30b, miR-222b, miR-320c in TNBC urine compared to HC urine.	[73]
11 TNBC, 11 HER2+, 24 LumA, 20 LumB, 16 HC/BC: 47 yrs, HC: 45 yrs	Stage I (10), stage II (31), stage III (25); all patients analyzed before surgery and NAC	Plasma (ctmiRNA)	ctmiRNA expression profile	miScript miRNA PCR array human cancer Pathway Finder kit; miScript SYBR Green PCR kit	Specific Mirnoma signature for each BC subtype: LumA (miR-29b dw, miR-155 up, miR-181c dw), LumB (miR-148a dw, let-7d up, miR-92a up, let-7b up, miR-15a dw), HER2+ (miR-125b up, miR-134 dw, miR-143 up, miR-135b dw) and TNBC (miR-17 up, miR-150 dw, miR-210 up, miR-372 dw, let-7f dw, miR-133b up, miR-146b up, miR-7 up).	[76]
72 TNBC, 105 non-TNBC, 60 BBD, 86 HC/TNBC: 46 < 50 yrs, 26 > 50 yrs, non-TNBC: 62 > 50 yrs, 43 > 50 yrs, BBD: 38 < 50 yrs, 22 > 50 yrs, HC: 53 < 50 yrs, 33 > 50 yrs	TNBC: stage I-II (42), stage III-IV (30); non-TNBC: stage I-II (59), stage III-IV (46)	Serum (ctlncRNA)	Expression of TINCR	qRT-PCR	TINCR is overexpressed in TNBC patients and is associated with worse clinicopathologic features than in other BC groups or controls (BBC; HC).	[81]
57 TNBC, 124 HC/17 TNBC and 32 HC: 20–44 yrs; 10 TNBC and 17 HC: 45–49 yrs; 7 TNBC and 14 HC: 50–54 yrs; 7 TNBC and 19 HC: 55–59 yrs; 3 TNBC and 7 HC: 60–64 yrs; 8 TNBC and 21 HC: 65–69 yrs; 5 TNBC and 14 HC: >70 yrs	Stage 0 (1), stage I (13), stage II (26), stage III (9), stage IV (2), unknown (6); relapsed (18), metastatic (2)	Plasma (ctlncRNA)	A panel of specific methylation from a discovery set	MethyLight droplet digital PCR (ddPCR)	LINC00299 is hypermethylated in TNBC compared to HC.	[82]

## 3. Conclusions

**Figure 2:**
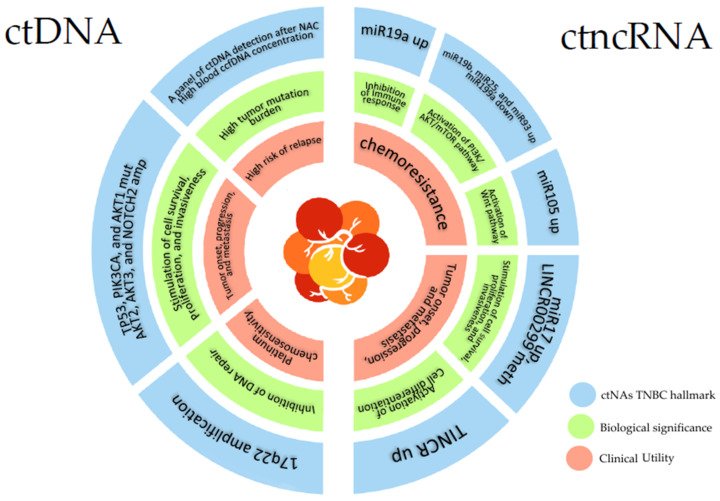
Schematic representation of the main ctNAs TNBC hallmarks described in this review. In the figure are reported also the biological significance and clinical consequences of each analyzed hallmark. Abbreviations: amp: Amplification; ccfDNA (circulating cell-free DNA); ctDNA: circulating cell-free tumor DNA; ctncRNA: circulating cell-free tumor non-coding RNA; down: downregulated; meth: hypermethylated; mut: mutated; up: upregulated.

Triple-negative breast cancer (TNBC) is an aggressive subtype of breast cancer. It is characterized by a great heterogeneity and absence of the HER2, ER, and PR receptors, which limits the number of possible therapies [6,7]. The late-stage diagnosis further complicates clinical outcomes [8]. The high mortality rate in TNBC highlights the need to find efficient biomarkers for molecular characterization, response to therapies, and prognosis. From this point of view, circulating cell-free tumor nucleic acids (ctNAs) may represent an interesting and non-invasive tool to pursue this goal [24,30,32]. The ctNAs can be found in body fluids in low concentration and highly fragmented, but the improvement of collection and characterization techniques such as ddPCR and NGS have increased the number of ctNAs analyses [33,34,35]. A total of 788 TNBC patients have been screened for ctNAs to date, and the results are very promising for their use in the clinic as informative molecules for follow-up, and therapeutic management. Both ctDNA and ctncRNA may be useful to predict chemoresistance and tumor progression, and give the oncologist time to plan the appropriate treatment for each patient. Figure 2 summarizes the main finding on the role of cfNAs in TNBC as biomarkers.

In TNBC, numerous reports have indicated that ctDNA fraction is an important biomarker for chemotherapy response and tumor progression. It is well known that a high level of ctDNA after neoadjuvant chemotherapy (NAC) is associated with shorter progression-free survival and relapse-free survival [38,39,40,41,42,43,48,49]. Similarly, a concentration of circulating cell-free DNA above 264 ng/mL is a strong marker of increased risk of relapse [40]. These parameters may be useful as independent prognostic biomarkers in TNBC because they represent the systemic tumor burden, including primary sites or metastases [40]. The detection of specific alterations in ctDNA has shown some important clinical implications. For example, TNBC patients who had 17q22 amplification had better progression-free survival after cisplatin treatment than those without amplification [55]. The cisplatin favoring effect of 17q22 amplification may be related to the overexpression of some genes in this region, such as KIF2B, TRIM37, NR1D1, and DLX4. Their overexpression leads to the inhibition of DNA repair and improves the sensitivity of TNBC to DNA-damaging platinum chemotherapy [51,52,53,54]. In addition, mutations and copy number variations of TP53 and PIK3CA/AKT genes in plasma have a high clinical significance. Mutations of TP53 and PIK3CA have been associated with TNBC progression events and chemotherapy resistance [42], while a higher amplification rate for AKT2 and AKT3 was found in the plasma of metastatic TNBC patients compared to paired TNBC patients with primary tumor [56]. These results are not unexpected, as dysregulation of TP53 and of the PIK3CA/AKT pathway is usually found in tumors and also in TNBC is a molecular footprint for onset, progression, and metastasis [21,22]. The demonstrated high concordance between ctDNA and genomic DNA mutations in tissues confirms the clinical value of genetic alterations detected in blood samples [42,43]. As for circulating ncRNAs, expression profiling of circulating tumor miRNA (ctmiRNA) has provided promising results for its use as a biomarker to characterize different breast cancer subtypes Moreover, these efforts have identified several miRNAs whose plasma dysregulation is an important predictive marker for chemoresistance in TNBC [64,67,72,73,76]. As expected, dysregulation of these miRNAs is associated with poor overall survival of TNBC patients [67,72]. In addition, overexpression of miR19a has been shown to inhibit anti-tumor immunity [65], while miR19b activates NFkB and suppresses PTEN, promoting cell proliferation and survival [68]. Overexpression of miR25 and miR93 stimulates activation of the AKT signaling pathway, leading to stimulation of cell survival, proliferation, and invasiveness [69,70]. Downregulation of miR199a is involved in the activation of mTOR and affects the sensitivity to doxorubicin [71]. Analysis of circulating cell-free tumor non-coding RNAs is not limited to miRNAs but also involves the long non-coding RNAs (lncRNAs). Overexpression in plasma samples of TINCR, a lncRNA involved in the regulation of cell proliferation and differentiation, has been associated with worse overall survival in TNBC patients [81]. Hypermethylation of circulating LINC00299 has been found to be specific for TNBC [82]. This lncRNA is involved in cell development, proliferation, and maintenance through the binding of various transcription factors, such as RAD21, EP300, GATA2, and GATA3 [83,84]. Despite these encouraging results, it is important to note the limitations of the clinical application of liquid biopsy. For example, the ability of ctNAs to detect patients with early-stage disease or in situ tumors remains uncertain. This is likely because ctNAs in body fluids do not reach detectable levels until a tumor develops at some mm size. For this reason, high-quality mammography remains the most important method for detecting early breast cancer [85]. Despite remarkable advances in methods to purify ctNAs, their low concentration in body fluids is still an important limitation for massive clinical use. Fortunately, increasingly efficient and sensible purification techniques are under development and optimization for ctNAs recovery. The possible methodological approaches are diverse and range from nanotechnology with the use of carbon nanotube transistors biosensors equipped with tetrahedral DNA nanostructures for ctDNA detection [86], to more classical techniques such as one-step branched rolling circle amplification for the measurement of ctmiRNA in serum samples [87]. In conclusion, the role of ctNAs as biomarkers in TNBC for clinical management seems to be solid and the available technologies are ready to introduce ctNAs in the clinic as another analysis to improve TNBC diagnosis, treatment, and prognosis.

## Figures and Tables

**Table 1:** Summary of clinical features of TNBC, experimental method, and main results in liquid biopsy on ctDNA. Abbreviations: cfDNA (circulating cell-free DNA); ctDNA (circulating cell-free tumor DNA); EFS (event-free survival); ER+ (estrogen receptor-positive breast cancer); HER2+ (HER2-positive breast cancer); HR+ (hormone receptor-positive breast cancer); MAF (mutant allele frequencies); NAC (neoadjuvant chemotherapy); OS (overall survival); PFS (progression-free survival); RFS (relapse-free survival); TNBC (triple-negative breast cancer); TFx (tumor fraction of cfDNA); VAF (variant allele frequencies); yrs (years). Staging refers to the first diagnosis.

Number of Patients and Controls/Age	Clinical Features at Sample Collection (Number of Patients)	Source of ctDNA	Target	Method	**Main Finding**	**Ref.**
13 TNBC, 11 HER2+, 20 ER+/57% patients < 50 yrs, 43% > 50 yrs	Stage II (31) and Stage III (14); all patients analyzed at pre- and post-NAC, pre- and post-surgical resection	Plasma	A panel of personalized tumor-informed DNA variants	bespoke multiplex polymerase chain reaction NGS ctDNA assay, Signater	Higher ctDNA detection at baseline (before NAC) in TNBC patients than in HER2+ and ER+. Shorter EFS for patients with high VAF after NAC time point and beyond.	[38]
72 TNBC/46 yrs (25–71)	Stage I (5), Stage II (43), Stage III (23), Stage IV (1), 18 relapsed; all patients analyzed pre- and post-NAC	Plasma	cfDNA concentration	SYBR Gold Nucleic Acid Gel Stain	The average cfDNA concentration decreased significantly after NAC. Patients with a cfDNA concentration > 264 ng/mL have a higher risk of relapse.	[40]
31 TNBC/19 patients < 50 yrs, 12 patients > 50 yrs	Stage II (24), Stage III (7), metastatic (9), relapsed (1); all patients analyzed before and after NAC	Plasma	A panel of personalized tumor-informed DNA mutations	IonAmpliSeq Cancer Hotspot Panel v2 CHPv2, ddPCR	The detection ctDNA after NAC resulted in shorter EFS.	[41]
89 TNBC/54 yrs (26–81)	All metastatic patients analyzed before and during treatment with Ipatasertib plus placitaxel	Plasma	cfDNA genomic profile	FoundationACT hybrid capture NGS assays, FoundationOne hybrid capture NGS assays	Patients with PIK3CA or AKT1 mutations had 100% concordance between ctDNA and tissue sequencing. Patients with PIK3CA/AKT mutations have a higher improvement in PFS after treatment with Ipatasertib than patients without mutations. High VAF on-treatment was associated with worse PFS.	[42]
22 TNBC, 44 HR+, 20 HER2+, (progression group, PG); 6 TNBC, 54 HR+, 2 HER2+, (control group, CG)/ 19 PG and 10 CG patients < 45 yrs, 51 PG and 36 CG patients: 45–65 yrs, 17 PG and 16 CG patients > 65 yrs	All metastatic patients analyzed at three different time points during the disease clinical management	Plasma	A panel of NCNN-recommended genes mutations and CNVs	Guardant360 NGS assay	Increase in MAF at different time points was associated with events of tumor progression. TP53, PIK3CA (for TNBC), ESR1, FGFR1, AR, and ERBB2 (for HR+) are key alterations associated with progression and chemotherapy resistance. The changes in TP53, PIK3CA, ERBB2, EGFR, ESR1, CCNE1, MYC, NF1, MET, and KIT showed high concordance (approximately 80%) between plasma and tissue.	[43]
25 TNBC, 29 HER2+, 41 ER+ 25 TNBC, 29 HER2+, 41 ER+/TNBC: 52 ± 10.2 yrs; HER2+: 49.3 ± 8.7 yrs; ER+: 49.2 ± 7.8 yrs	Stage II and III patients analyzed before and after NAC-surgery	Plasma	TP53, PI3KCA, HER2, GATA3, CDH1, PTEN, AKT1, ESR1, S100A7-9, ZNF703, B2M, CCND1, c-MYC mutations and CNVs	QIAseq Targeted DNAPanel, Illumina MiSeq Reagent Kit v2, 2 × 150 bp reads, OncoCNV	Detection of ctDNA after NAC led to short RFS. The RFS of TNBC patients was shorter than in HER2+ patients.	[48]
26 TNBC/15 patients < 50 yrs; 11 patients > 50 yrs	Unknown (2), Stage I (1), Stage II (19), Stage III (4); all patients analyzed before, during, and after NAC	Plasma	A personalized panel of 4–5 tumor-informed variants	Whole exome sequencing (WES), ddPCR	ctDNA detection during NAC was strongly predictive of residual tumor at the surgery. ctDNA detection at the end of NAC indicated significantly worse relapse-free survival and overall survival.	[49]
58 TNBC/45 yrs	Stage I (12), Stage II (33), Stage III (17), Stage IV (8); all patients are treated with cisplatin alone or in combination with paclitaxel	Plasma	amplification of 17q22	Gistic 2.0	Patients with 17q22 amplification have a better PFS after cisplatin treatment.	[55]
164 TNBC/34 patients < 40 yrs, 62 patients: 40–50 years, 45 patients: 50–60 yrs, 20 patients > 60 yrs	Stage I (22), Stage II (80), Stage III (43), Stage IV (16); all metastatic patients received NAC treatment	Plasma	cfDNA genomic profile, CN of 25 breast cancer-related genes	Low coverage whole genomic sequencing	TFx > 10% was associated with significantly worse OS. A higher amplification rate for AKT2, AKT3, and NOTCH2 was seen in metastasis compared to paired primary tumors.	[56]
15 TNBC/48 yrs	Early-stage patients analyzed after NAC and before surgical resection	Plasma; urine	A panel of 93 breast cancer-related genes mutations	QIAseq Human Breast cancer PaneL, Illumina NGS	Mutations of NF1, CHEK2, KMT2C, and PTEN shown in paired blood and urine biopsy.	[57]

## Data Availability

Not applicable.

## Abbreviations

BLIS	Basal-like triple-negative breast cancer
ccfDNAs	Circulating cell-free DNAs
ccfNAs	Circulating cell-free nucleic acids
cfDI	Circulating cell-free DNA integrity
CTCs	Circulating tumor cells
ctDNAs	Circulating cell-free tumor DNAs
ctmiRNAs	Circulating cell-free tumor microRNAs
ctmRNAs	Circulating cell-free tumor messenger RNAs
ctNAs	Circulating cell-free tumor nucleic acids
ctncRNAs	Circulating cell-free tumor non-coding RNAs
ER+	Estrogen receptor-positive breast cancer
HER2+	HER2-positive breast cancer
HR+	Hormone receptor-positive breast cancer
IM	Immuno modulatory triple-negative breast cancer
LAR	Luminal androgen receptor triple-negative breast cancer
MAF	Mutant allele frequencies
MES	Mesenchymal-like triple-negative breast cancer
NAC	Neo-adjuvant chemotherapy
PARPi	PARP inhibitor
PR+	Progesterone receptor-positive breast cancer
TFx	Tumor fraction of circulating cell-free DNA
TNBC	Triple-negative breast cancer
tncRNAs	Tumor non-coding RNAs
VAF	Variant allele frequencies
